# Association between the -844 G>A, HindIII C>G, and 4G/5G *PAI-1* Polymorphisms and Susceptibility to Multiple Sclerosis in Western Mexican Population

**DOI:** 10.1155/2019/9626289

**Published:** 2019-10-07

**Authors:** Emmanuel Valdés-Alvarado, Miguel Huerta, Xochil Trujillo, Yeminia Valle, Jorge Ramón Padilla Gutiérrez, Mario Alberto Mireles-Ramírez, Miguel Angel Macías-Islas, Baltazar-Rodríguez Luz Margarita, José Francisco Muñoz-Valle

**Affiliations:** ^1^Instituto de Investigación en Ciencias Biomédicas, Centro Universitario de Ciencias de la Salud, Universidad de Guadalajara, Guadalajara, Jalisco 44340, Mexico; ^2^Centro Universitario de Investigación Biomédica, Universidad de Colima, Colima, Colima 28040, Mexico; ^3^Unidad Médica de Alta Especialidad (UMAE), Hospital de Especialidades, Centro Médico Nacional de Occidente, IMSS, Guadalajara, Jalisco 4430, Mexico; ^4^Doctorado en Farmacología, Centro Universitario de Ciencias de la Salud, Universidad de Guadalajara, Guadalajara, Jalisco 44340, Mexico

## Abstract

**Introduction:**

Multiple sclerosis is an inflammatory disease, where fibrin deposition and the impairment in its degradation have been shown to play an important role in the demyelination process. Tissue plasminogen activator (tPA) is a serine protease that enhances the conversion of plasminogen into its active form plasmin, the principal tPA inhibitor is the PAI-1. Several *PAI-1* polymorphisms impact its gene expression and protein activity. Furthermore, the aim of this study was to investigate the association between the - 844 G>A, HindIII C>G, and 4G/5G *PAI-1* polymorphisms and susceptibility to MS.

**Material and Methods:**

The study group included 400 Mexican mestizo subjects: 200 unrelated patients and 200 unrelated individuals identified as control subjects. The analysis of *PAI-1* polymorphisms was performed by polymerase chain reaction-restriction fragment length polymorphism.

**Results:**

A significant association was found between the CG genotype of the *HindIII* C>G *PAI-1* polymorphism and susceptibility to MS (OR = 1.58, *p* = 0.03); moreover, the frequency of 5G allele and 5G/5G genotype of the 4G/5G *PAI-1* polymorphism was statistically significant (OR = 1.36 and *p* = 0.04 and OR = 2.43 and *p* = 0.02, respectively). With respect to the relation between the scores of progression (EDSS) and severity (MSSS), no association was found between EDSS and genotypes of the *PAI-1* polymorphisms analyzed. Regarding MSSS, male that carries genotype GA of the -844 G>A and genotype 4G/5G of the 4G/5G *PAI-1* polymorphisms showed a significant association with an increase of media of MSSS in comparison with females (*p* = 0.01 in both cases).

## 1. Introduction

Multiple sclerosis (MS) is an autoimmune inflammatory disease, affecting the myelin sheath in the central nervous system [1]. The Multiple Sclerosis International Federation (MSIF), on their last Atlas study published in 2013, estimates a pooled prevalence of 29/100,000 [2] while in Mexico is estimated to range from 11 to 20 cases per 100,000 inhabitants [3]. This disease has a lifetime risk of one in 400, leading it to be one of the most common causes of nontraumatic neurological disability in young adults [4]. Although the event that triggers the onset of MS is not clear, the evidence suggests that a strong interaction between the genomics and the environment is the main component of the disease [1]. In recent years, fibrin deposition and the impairment in its degradation have been shown to play an important role in the demyelination process, which precedes the onset of clinical signs [5]. Tissue plasminogen activator (tPA) is a serine protease that enhances the conversion of plasminogen into its active form plasmin [1, 5]. Plasmin is a major enzyme for fibrin degradation; therefore, tPA entails straight fibrinolysis regulation [6]. The principal tPA inhibitor is the plasminogen activator inhibitor 1 (PAI-1); this protein's gene is located on chromosome 7 q21.3-22 and consists of 9 exons and 8 introns. The main regulator of PAI-1 expression is the transforming growth factor TGF-*β* [6, 7]. It has been described that several *PAI-1* polymorphisms impact in gene expression and protein activity; including the -675 4G/5G polymorphism where the individuals with the allele 4G present higher PAI-1 levels due to the lack of a binding site for a transcription repressor. Similarly, the gene transcription may be influenced by a nucleotide substitution of G>A in the -844 position affecting the binding of nuclear factors due to an alteration in the consensus sequence. Finally, the HindIII polymorphism C>G has been also associated with high PAI-1 levels in individuals with the GG genotype [8, 9]. Altered PAI-1 levels observed in animal models have been associated with a diverse spectrum of diseases, low levels correlate with accelerated atherosclerosis and a defect in local angiogenesis; in counterpart, higher PAI-1 levels are found to be produced by malignant cells leading to a hypercoagulation state and in multiple sclerosis tissue interfering with fibrin degradation and contributing to axonal damage [5, 7]. HindIII polymorphism has been already studied in other disease such as systemic lupus erythematosus (SLE), recurrent miscarriages, and risk of ischemic stroke; but it has never been associated with multiple sclerosis [9, 10]. Based on this knowledge, the aim of this study was to investigate the association between the -844 G>A, HindIII C>G, and 4G/5G *PAI-1* polymorphisms and susceptibility to MS in western Mexican population.

## 2. Materials and Methods

### 2.1. Subjects

The study group included 400 Mexican mestizo subjects: 200 MS unrelated patients diagnosed by a neurologist according to the “McDonald Criteria for Multiple Sclerosis” [11] recruited from the “Centro Medico Nacional de Occidente” and the “Hospital Civil Fray Antonio Alcalde” in Guadalajara city, Mexico, and 200 unrelated individuals identified as control subjects (CS) and age- and sex-matched with MS patients. We considered Mexican mestizo subjects, only those individuals who for three generations, including their own, had been born in western Mexico. The Expanded Disability Status Scale (EDSS) and the Multiple Sclerosis Severity Score (MSSS) were applied to the patients either during their recruitment or 3 months later if they were recruited during a clinical relapse [12].

### 2.2. Ethics Statement

The study was performed according to the ethical principles for experiments involving humans stated on the Declaration of Helsinki, and ethical approval was obtained by the Centro Universitario de Ciencias de la Salud, CUCS, UdeG. Informed consent was obtained from all patients for being included in the study.

### 2.3. Genotyping of the -844 G>A, HindIII C>G, and 4G/5G PAI-1 Polymorphisms

Genomic DNA was extracted from peripheral blood leukocytes using Miller's Technique [13]. The analysis of -844 G>A, HindIII C>G, and 4G/5G *PAI-1* polymorphisms was analyzed by polymerase chain reaction-restriction fragment length polymorphism (PCR–RFLP) as previously described [9, 10, 14].

### 2.4. Statistical Analysis

The Hardy-Weinberg equilibrium test, genotype, and allele frequencies were calculated by the *χ*^2^ or Fisher's exact test, when applicable. Odds ratios (OR) and 95% confidence intervals (95% CI) were calculated to test the probability that the genotype and allele frequencies were associated with MS. A *p* value < 0.05 was considered to be statistically significant. All the statistical analyses were done with the SPSS statistical package version 20. The sample size was calculated according to the Kelsey formula for proportions in case control studies ([15]). The association of EDSS, MSSS, and gender with the *PAI-1* genotypes was determinate by the Mann-Whitney *U* or Kruskal-Wallis test. Bonferroni's correction was applied to reduce the statistical type 1 error (*p*_c_) in multiple comparisons.

## 3. Results

### 3.1. Clinical and Demographic Characteristics

All clinical characteristics are shown in Table 1. The median age of the CS and MS groups was 36 (28-44) and 35 (28-43) years, respectively. The gender distribution among MS patients was 71% female and 21% male. The MS individuals had a median disease duration of 8 years; also, these patients showed a median of 1.9 of EDSS and a median of 3.7 of MSSS. Regarding the clinical variant, 94% were recurrent remittent multiple sclerosis (RRMS) and 6% were secondary progressive multiple sclerosis (SPMS). 51% of patients were treated with glatiramer acetate.

### 3.2. Analysis of the -844 G>A, HindIII C>G, and 4G/5G PAI-1 Polymorphisms

No deviation from the Hardy-Weinberg equilibrium was detected in the -844 G>A, HindIII C>G, and 4G/5G *PAI-1* polymorphisms (*p* = 0.79, 0.07, and 0.11, respectively). The allele and genotype frequencies in MS individuals and CS are shown in Table 2. The comparisons of genotype and allele frequencies for the -844 G>A *PAI-1* polymorphism between both study groups did not show significant differences. However, a significant association was found between the CG genotype of the *HindIII* C>G *PAI-1* polymorphism and susceptibility to MS (47% versus 34% in MS and CS, respectively, *p* = 0.03). With respect to the analysis of genotype and allele frequencies of the 4G/5G *PAI-1* polymorphisms, a significant difference for the 5G allele frequency (33% versus 30.5% in MS and CS, respectively, *p* = 0.04) and 5G/5G genotype frequency (11.5% vs. 5.5% in MS and CS, respectively, *p* = 0.02) was observed.

### 3.3. Association between EDSS, MSSS, PAI-1 Genotypes, and Gender

EDSS and MSSS are two approaches to measure differences in the progression of MS. For that reason, we stratified both scores and tested the relation with respect to alleles, genotypes of *PAI-1* polymorphisms, and gender (Table 3). No differences were found between alleles of the *PAI-1* polymorphisms and EDSS or MSSS (data not shown). Males with GG genotype of -844 G>A *PAI-1* polymorphism showed higher levels in the median of EDSS in comparison with females (3.9 vs. 2.7) (*p* = 0.04). In the same manner, male that carries genotype 4G/5G of the 4G/5G PAI-1 polymorphism showed higher media of EDSS with respect to female (3.3 vs. 2.3) (*p* = 0.03). With respect to MSSS, male that carries genotype GA of the -844 G>A *PAI-1* polymorphism, genotypes CC and CG of the HindIII C>G *PAI-1* polymorphism, and genotypes 4G/4G and 4G/5G of the 4G/5G *PAI-1* polymorphism showed an increase of media of MSSS in comparison with females ((5 vs. 3.4 (*p* = 0.01), 5.1 vs. 3.5 (*p* = 0.02), and 5.4 vs. 3.9 (*p* = 0.04), respectively) and (5.6 vs. 4 (*p* = 0.03) and 5.1 vs. 3.3 (*p* = 0.01), respectively)). However, after correction, values > 0.017 lacked statistical significance.

## 4. Discussion

MS is a chronic demyelinating disease of the central nervous system, characterized as a complex disease due to several causes that underlie its development and are still incompletely understood [16]. Age and gender are risk factors that predispose to the development of MS; in our study group, the mean age and the proportion of female/male are consistent with previous reports [1, 17]. Otherwise, studies of the molecular basis underlying the pathogenesis of MS are an important part of research in the field, helping to design new strategies for its prevention and treatment; in this sense, the fibrinolytic system components are key molecules in the explanation of cognitive impairment in MS [1]. PAI-1 is a key molecule in the fibrinolytic system; its functions are related to the inhibition of the plasminogen activator which blocks the conversion of plasminogen to plasmin. *PAI-1* polymorphisms increase or decrease plasma concentrations of PAI-1; to date, only three studies have investigated the correlation between these polymorphisms and MS [1, 18, 19]. We analyzed the -844 G>A, HindIII C>G, and 4G/5G *PAI-1* polymorphisms. The comparisons of allele and genotype frequencies for the -844 G>A polymorphism between both study groups did not show significant differences. The allelic and genotypic frequencies found of the *PAI-1* polymorphism are similar in previous studies in the Mexican population [6, 9, 10]. The -844 G>A polymorphisms have been associated with several diseases, including venous thrombosis and ischemic stroke caused by small vessel disease [8, 20]. In previous studies in the Mexican population, García-González et al. found an association of the A allele and AA genotype with acute coronary syndrome ([9]); meanwhile, Padilla-Gutiérrez et al. described a significant difference of the A allele in SLE patients [10]. It is already known that higher levels of PAI-1 could confer a risk for the development of cardiovascular disease and SLE; moreover, different reports supported the relationship existing between the A allele and AA genotype with superior amounts of protein with respect to MS; high levels of PAI-1 could be associated with a good outcome; this could be an explanation why we did not find an association between this polymorphism and MS. Regarding the HindIII C>G polymorphism, a significant association was found between the CG genotype and susceptibility to MS (OR = 1.58, *p* = 0.03). To the best of our knowledge, HindIII C>G polymorphism has not been studied in MS so far; notwithstanding, the GG genotype has been associated with high plasma PAI-1 level [8]. In this sense, the association of the GC found in our research could be related with decreased PAI-1 levels. With respect to 4G/5G *PAI-1* polymorphisms, a significant difference for the 5G allele (OR = 1.36, *p* = 0.04) and 5G/5G genotype (OR = 1.43, *p* = 0.02) was observed. In particular, this genetic variant has been studied in several diseases with different results. The 4G/4G genotype was associated with the decreased risk of cerebrovascular mortality, but there is possible protective effect of 4G/4G against stroke of unknown etiology [8]; otherwise, the 4G allele has been found to have opposite effects in cardiac disease (increased risk) compared to brain ischemia (protective effect) [8, 21]. Specifically in MS, this polymorphism has been analyzed in different populations. Zivković et al. reported the *PAI* 5G5G genotype as a risk factor for MS pooled (Serbian, Bosnian and Herzegovinian, Croatian, and Slovenian) patients ([1]); Luomala et al. found that the 5G5G genotype was associated with MS in Finnish women, and Lovrecic et al. describe a borderline significance of TPA DD/PAI-1 4G4G genotype combination for reduced risk for MS Slovenian and Croatian patients [18]. All these results accord with our results; as is well described, the 5G5G genotype is linked with lower plasma levels of PAI-1. Moreover, the 5G allele binds a repressor protein to an overlapping site, which leads to a decreased basal level of PAI-1 transcription; otherwise, haplotypes containing the PAI-1 4G allele have been associated with higher transcriptional activity in astrocytes compared to haplotypes containing 5G which could perform a neuroprotective role of sufficient PAI-1 in the brain through allele-specific regulation of its presence ([19]; P. [22]). Accordingly, with the interaction between EDSS, MSSS, PAI-1 genotypes, and gender, we found that males that carry GA and 4G5G genotypes showed an increase of media of MSSS. These results differ from a study published by Luomala et al. who found an association with the 5G5G genotype and MS in women. It is already documented that estrogens diminish plasma concentrations of PAI-1 [19] and also that the *PAI-1* polymorphism alter its plasma concentration; nevertheless, it is important to highlight that PAI-1 has demonstrated a mechanism responsible for modulation of the immune response, which suggests a polarization in the T cell response [10]; so we hypothesized that the alteration of genotypes of the production of PAI-1 in combination with the rate expression of sex hormones could be related to the severity and progression of the disease. However, future studies are necessary to clarify the relationship and the mechanisms involved.

## 5. Conclusion

In conclusion, we found an association of the CG genotype, 5G5G genotype, and 5G allele of the HindIII C>G and 4G/5G *PAI-1* polymorphisms, respectively, with MS patients from western Mexico. According to the severity (EDSS), genotypes of -844 G>A, HindIII, and 4G/5G *PAI-1* polymorphisms are not related to higher scores in MS patients. Nevertheless, males that carry the GA genotype of the -844 G>A, CC, and 4G4G and 4G5G genotypes of the 4G/5G *PAI-1* polymorphisms are associated with an increased risk of progression (MSSS) of the disease.

## Figures and Tables

**Table 1:** Clinical and demographic characteristics of MS patients and control subjects.

Variable	MS (*n* = 200)	CS (*n* = 200)
Demographics		
Age (years)^1^	35 (28-43)	36 (28-44)
Gender^2^		
Male	29 (58)	30 (60)
Female	71 (142)	70 (140)
Clinical characteristics		
Disease duration (years)^1^	8	—
Disease severity^1^		
EDSS	1.9 (0.9-4.4)	—
MSSS	3.7 (1.1-6.5)	—
Clinical variant^2^		
RRMS	94 (188)	—
SPMS	6 (12)	—
Therapy^2^		
Treatment naive	11 (22)	—
Glatiramer acetate	51 (102)	—
Beta interferons	23 (46)	—
Azathioprine	3 (6)	—
Biologicals (natalizumab and fingolimod)	0.5 (1)	—
Combined immunosuppressive therapy	3 (6)	—

EDSS = expanded disability status scale; MSSS = multiple sclerosis severity score; RRMS = recurrent remittent multiple sclerosis; SPMS = secondary progressive multiple sclerosis. ^1^Data is shown in median (p25-p75). ^2^Data is shown in percentage.

**Table 2:** Allele and genotype distributions of -844 G>A, HindIII C>G, and 4G/5G *PAI-1* polymorphisms in MS and CS.

Polymorphism	MS % (*n* = 200)	CS % (*n* = 200)	OR (CI 95%); *p*^∗^
-844 G>A			
Genotype			
GG	40 (80)	45.5 (91)	1
GA	52.5 (105)	44.5 (89)	1.3 (0.88-2.02); 0.16
AA	7.5 (15)	10 (20)	0.8 (0.41-1.77); 0.67
Allele			
G	33.8 (135)	67.8 (271)	1
A	66.2 (265)	32.2 (129)	1.1 (0.79-1.43); 0.65
HindIII C>G			
Genotype			
CC	44 (88)	50.5 (101)	1
CG	47 (94)	34 (68)	**1.58 (1.04-2.42); 0.03**
GG	9 (18)	10.5 (21)	0.98 (0.49-1.964); 0.96
Allele			
G	32.5 (130)	30 (120)	1.12 (0.833-1.515); 0.44
C	67.5 (270)	70 (280)	1
4G/5G			
Genotype			
4G/4G	45.5 (91)	44.5 (89)	1.18 (0.79-1.79); 0.40
4G/5G	43 (86)	50 (100)	1
5G/5G	11.5 (23)	5.5 (11)	**2.43 (1.12-5.27); 0.02**
Allele			
4G	67 (268)	69.5 (278)	1
5G	33 (132)	30.5 (122)	**1.36 (1.01-1.83); 0.04**

MS: multiple sclerosis; CS: control subjects; OR: odds ratio; CI: confidence interval. ^∗^*p* < 0.05.

**Table 3:** Association between EDSS, MSSS, *PAI-1* genotypes, and gender.

Polymorphism	EDSS				MSSS		
	Female (*n* = 142)	Male (*n* = 58)	*p*		Female (*n* = 142)	Male (*n* = 58)	*p*
-844 G>A							
GG	2.7 ± 2.2	3.9 ± 2.3	0.04	GG	4.2 ± 3.1	5.5 ± 3.3	0.09
GA	2.7 ± 2.2	3.2 ± 2	0.26	GA	3.4 ± 2.9	5 ± 2.8	**0.01**
AA	1.9 ± 2.1	2.8 ± 3	0.58	AA	3.1 ± 3	5.3 ± 4.5	0.34
HindIII C>G							
CC	2.5 ± 2.1	3.4 ± 2.1	0.06	CC	3.5 ± 2.9	5.1 ± 2.9	0.02
CG	2.7 ± 2.3	3.5 ± 2.3	0.12	CG	3.9 ± 3.1	5.4 ± 3.2	0.04
GG	2.8 ± 1.9	2.9 ± 2.4	0.94	GG	4.2 ± 2.5	4.6 ± 3.2	0.77
4G/5G							
4G/4G	2.8 ± 2.2	3.5 ± 2.4	0.21	4G/4G	4 ± 3.1	5.6 ± 3.3	0.03
4G/5G	2.3 ± 2	3.3 ± 2	0.03	4G/5G	3.3 ± 2.8	5.1 ± 2.8	**0.01**
5G/5G	2.8 ± 2.2	3.8 ± 2.7	0.39	5G/5G	4 ± 3	4.3 ± 3.2	0.85

Data is provided as median (±DS) ^∗^*p* values Mann-Whitney *U* test. Corrected significance level *p*_c_ < 0.017.

## Data Availability

The data used to support the findings of this study are included within the article.
